# An integrated analysis revealed different microRNA-mRNA profiles during skeletal muscle development between Landrace and Lantang pigs

**DOI:** 10.1038/s41598-017-02558-7

**Published:** 2017-05-31

**Authors:** Shuihua Xie, Luxi Chen, Xumeng Zhang, Xiaohong Liu, Yaosheng Chen, Delin Mo

**Affiliations:** 1 State Key Laboratory of Biocontrol, Sun Yat-sen University, Guangzhou, 510006 Guangdong China; 2 General Station of Animal Husbandry Technology Extension, Department of Agriculture of Guangdong Province, Guangzhou, 510500 Guangdong China

## Abstract

Pigs supply vital dietary proteins for human consumption, and their economic value depends largely on muscle production. MicroRNAs are known to play important roles in skeletal muscle development. However, their relationship to distinct muscle production between pig breeds remains unknown. Here, we performed an integrated analysis of microRNA-mRNA expression profiles for Landrace (LR, lean) pigs and the Chinese indigenous Lantang pig (LT, lard-type) during 8 stages of skeletal muscle developmental, including at 35, 49, 63, 77 dpc (days post coitum) and 2, 28, 90, 180 dpn (days postnatal). As differentially expressed-miRNA expression profiles can be well classified into two clusters by PCA analysis, we grouped the embryonic stages as G1 and the postnatal stages as G2. A total of 203 genes were predicted miRNA targets, and a STEM analysis showed distinct expression patterns between G1 and G2 in both breeds based on their transcriptomic data. Furthermore, a STRING analysis predicted interactions between 22 genes and 35 miRNAs, including some crucial myogenic factors and myofibrillar genes. Thus, it can be reasonably speculated that myogenic miRNAs may regulate myofibrillar genes in myofiber formation during embryonic stages and muscle hypertrophy during postnatal stages, leading to distinct differences in muscle production between breeds.

## Introduction

Pigs supply vital dietary proteins for human consumption, and their economic value depends largely on muscle production. Previous research has shown that lean and lard-type pig breeds have significant genetic differences in terms of muscle growth rate and gene expression profiles^1^. We performed a miRNAome study and found that early muscle development was strongly correlated to miRNA expression in LR^2^. However, few recent studies have performed integrated analyses of miRNA-mRNA expression profiles in pig breeds with different muscle production. Landrace (LR), an improved commercial pig breed, is characterized by a high percentage of lean meat percentage, fast growing muscle tissue and high body weight^3–5^. In contrast, Lantang (LT), a pig breed indigenous to China, is characterized by a low percentage of lean meat, slow-growing muscle tissue and low body weight^3, 6^. The differences in the muscle production of LR and LT may therefore be a proper model for studying the mechanism underlying muscle development.

In pigs, muscle growth is predominantly determined during prenatal skeletal muscle development; the formation of primary myofibers occurs from 35 to 55 dpc, which is followed by the formation of secondary myofibers around each primary myofiber between 50 and 90 dpc^7–9^. Between late gestation to the first four postnatal weeks, myofibers undergo a maturation process^1, 8^. Hypertrophy is also a process of skeletal muscle maturation and is characterized by increased muscle mass, myofiber size, and myofibrillar protein content^10^.

miRNAs are endogenous small non-coding RNAs (~22 nucleotides) that modulate gene expression at the post-transcriptional level by binding to the 3′ untranslated region (3′-UTR) of target mRNAs^11^. Most miRNAs are evolutionarily conserved in related species, and recent studies have shown that some miRNAs are involved in regulating a variety of developmental and physiological processes^12^. Moreover, numerous miRNAs have been shown to be associated with skeletal muscle development. A recent study compared the pre- and post-natal microRNA expression profiles in the longissimus dorsi muscles of LR and Pietrain pigs, as the muscularity of this muscle differs between these breeds; those authors found that the dynamic expression and breed-associated regulation of porcine muscle miRNAs suggests a functional role for miRNA-mediated gene regulation during muscle development and in establishing phenotypic variations of muscle traits^13^. Our previous research discovered 18 novel candidate myogenic miRNAs in LR during muscle development^2^. Furthermore, our previous analysis of the transcriptome during skeletal muscle development between LR and LT identified 595 differentially expressed myogenic genes^1^. However, these findings are not sufficient to establish a comprehensive understanding of the relationships between miRNAs and mRNAs that regulate distinct muscle production between pig breeds. Thus, further research is required.

In this study, we performed an integrative analysis of the miRNA-mRNA expression profiles in LR and LT pigs during 8 stages of skeletal muscle development, including four prenatal stages (35, 49, 63, and 77 dpc) and four postnatal stages (2, 28, 90, and 180 dpn) using Solexa sequencing. Our study contributes to the understanding of the mechanisms that regulate muscle development and hypertrophy in pig breeds with distinct differences in muscle production.

## Materials and Methods

### Ethics Statement

The experiments were all carried out according to the Chinese Council on Animal Care, and the protocols conducted were approved by the Animal Care and Use Committee of Guangdong Province, China. The approval ID/permit numbers are SCXK (Guangdong) 2011–0029 and SYXK (Guangdong) 2011–0112.

### Sample collection

Thirteen purebred LR or LT sows with the same genetic background were artificially inseminated with the semen of the same purebred boar, respectively. For the prenatal stages 2 sows of each breed per time point were slaughtered at embryonic days 35, 49, 63, and 77, and the longissimus dorsi muscles of the fetuses were collected. For the postnatal stages, 3 gilts of each breed per time point were slaughtered at 2, 28, 90, and 180 days after birth, and muscle tissues from the same area of the longissimus dorsi were used as the experimental samples. All samples were snap-frozen in liquid nitrogen and stored at −80 °C.

### Small RNA sequencing and data analysis

We used a single pooling strategy (three samples per stage per pig breed were pooled before the libraries were constructed) in our study. Total RNA was extracted using a miRNeasy Mini Kit (QIAGEN, GmBH, Germany) according to the manufacturer’s protocol. At each stage, equal quantities of total RNA were isolated from three individual fetuses/pigs and pooled. The total RNA integrity was measured using an Agilent 2100 Bioanalyzer system (Agilent, CA, USA). RNA fragments of 16–35 nt were excised, purified from a PAGE gel, and ligated with 5′ and 3′ adaptors by T4 RNA ligase. Reverse transcription followed by PCR was used to build cDNA constructs based on the small RNA ligated with 3′ and 5′ adapters. Then, the amplified cDNA constructs were purified from agarose gel for sequencing analysis using an Illumina Genome Analyzer (Illumina, CA, USA) according to the manufacturer’s instructions.

The raw data were processed using the Illumina Genome Analyzer Pipeline software and submitted to data filtration. After filtering the low-quality reads and trimming the adaptor sequences, clean reads were obtained. We used the genomic position information of small RNAs and repeats in the annotation files from the UCSC Genome Browser and fRNAdb (http://www.ncrna.org/frnadb) to assign the mapped reads to RNA classes. For known miRNAs (miRBase ver. 18), the number of raw, clean tags in each sample was normalized to Tags per Million (TPM) to obtain the normalized gene expression levels. For novel miRNA discovery, we used the mirDeep2 software (version 2.0.5) with the default parameters. Unmappable reads were annotated and classified by reference to non-coding RNAs in the Ensemble (ftp://ftp.ensembl.org/pub/release-69/fasta/sus_scrofa/ncrna/), piRNA (http://pirnabank.ibab.ac.in/) and Rfam (version 10; http://rfam.sanger.ac.uk/) databases. Mappable sequences were mapped and used for further analysis. Previously submitted raw data (NCBI Sequence Read Archive (http://www.ncbi.nlm.nih.gov/Traces/sra/) under accession No. SRA073195) were utilized for LR analysis. Newly submitted raw data (NCBI Sequence Read Archive (http://www.ncbi.nlm.nih.gov/Traces/sra/) under accession SRR4409159) were utilized for LT analysis.

### RNA-seq data analysis

RNA sequencing data obtained from the same tissues as those used in miRNA sequencing (Gene Expression Omnibus (GEO) under series GSE25406)^1^ were utilized. The reference genome index was built using the Bowtie2-build component of Bowtie2 (ver. 2.0) and SAMtools (ver. 0.1.18). Tophat2 (version 2.0.8) was applied to map the reads to the reference genome. The expression levels of each gene were normalized as FPKM (Reads per kilobase of exon model per million mapped reads) using the Refseq gene (mm10) model downloaded from the UCSC Browser gateway. Each FPKM was log2-transformed.

### Determination of DE miRNAs

To compare the differential expression of miRNA in all samples between and within breeds during different stages of muscle development, we used TPM (transcripts per million mapped reads) to obtain normalized gene expression levels. The differential expression of miRNA or tags across samples was determined according to previously described methods^2^. miRNAs with a P-value ≤ 0.005, false discovery rate (FDR) ≤ 0.01 and estimated absolute log2-fold change ≥ 0.5 in sequence counts across libraries were considered to be significantly differentially expressed. To identify differentially expressed miRNAs across samples or groups the formula was shown as follows:

$$p(x|y)={(\frac{{N}_{2}}{{N}_{1}})}^{y}\frac{(x+y)!}{x!y!{(1+\frac{{N}_{2}}{{N}_{1}})}^{x+y+1}}$$

$$C(y\le {y}_{min}|x)=\sum _{y=0}^{y\le {y}_{min}}p(y|x)$$

$$D(y\ge {y}_{max}|x)=\sum _{y\ge {y}_{max}}^{\infty }p(y|x)$$

FDR is adjusted P-value and was calculated by multiplying the p value by the total number and then dividing the product by rank. In each comparison, we identified miRNAs with a fold change ≥2 and a P-value < 0.05 as significantly DE miRNAs. The statistical analyses were performed using SPSS 10.0.

### Principal components (PCA) analysis

To explore the similarity of these samples based on their miRNA expression pattern, the DE-miRNAs were used under the aforementioned criterion. All statistical analyses were performed in the R statistical program, version 2.9.2. PCA analysis was carried out using the R Companion to Applied Regression in R (http://cran.r-project.org/web/packages/car/index.html)^14^.

### STEM analysis

We performed STEM analysis to visualize the expression patterns of mRNAs using the Short Time-series Expression Miner (v 1.1, STEM) program^15^. Each mRNA was assigned to the model profile that its time series most closely matched based on the correlation coefficient. We then computed the number of mRNAs assigned to each model profile. The number of mRNAs expected to be assigned to a profile was calculated by randomly permuting the original time point values, renormalizing the mRNA expression values, assigning mRNAs to their most closely matching model profiles, and repeating this process for a large number of permutations. The average number of mRNAs assigned to a model profile over all permutations was used as the estimate of the expected number of mRNAs assigned to the profile. We also computed the statistical significance of the number of mRNAs assigned to each profile compared with the expected number.

### GO and statistical analysis

The biological process (BP) of each DE miRNA was annotated by the Blast2GO software (http://www.blast2go.org/). GO functional classification and enrichment analysis were also conducted to identify GO terms that were significantly enriched in DE miRNAs using DAVID analysis (http://david.abcc.ncifcrf.gov/). Heatmaps were drawn for the DE miRNAs using the R language package “Pheatmap.” The myogenic DEGs and predicted gene interaction network was drawn using the STRING (http://string-db.org/) and Cytoscape 3.1.0 (http://www.cytoscape.org/) programs. The P-values were determined using T-tests. The statistical analyses were conducted with SPSS 10.0.

### Validation of sequencing data

The miRNA data in this manuscript originated from 16 libraries consist of 8 LR and 8 LT skeletal muscle samples, and the 16 libraries were constructed by same methodology and were sequenced simultaneously. To validate the sequencing results, nine miRNAs with different expression levels were selected and Stem–loop RT-PCR was used as microRNA quantitative methodology, which performed using the Lightcycler480 (Roche) with SYBR-Green detection (SYBR PrimeScript RT-PCR Kit, TaKaRa Biotechnology Co. Ltd.) according to the manufacturer’s instructions. As a result, the Pearson correlation coefficient of the Stem–loop RT-PCR and the Solexa sequencing was calculated and the r values ranged from 0.84 to 0.95 across 8 development stages, indicating that there was a high consistency between the two methodologies. This validation assay has been performed in our previous published paper^2^.

The transcriptomic data has been verified using quantitative PCR approaches by our research group^1^. In which, nine genes with different expression level were chosen for real-time quantitative PCR analysis. Spearman’s correlation coefficient (r) indicated that the r values ranged from 0.76 to 0.96.

## Results

### The embryonic and postnatal stages were well separated by their miRNA expression profiles

After trimming of the adaptor sequences and removal of low quality reads, a total of 181,224,007 clean reads were identified, of which almost 96% matched the small RNA databases mentioned in the Materials and Methods. Based on the criteria of a P-value ≤ 0.005, FDR ≤ 0.01, and an estimated absolute log2-fold change ≥ 0.5 in sequence counts across libraries, 258 miRNAs were found expressed in longissimus dorsi muscle during all stages, of which 152 significantly DE-miRNAs were identified between LR and LT (Table S1).

**Figure 1:**
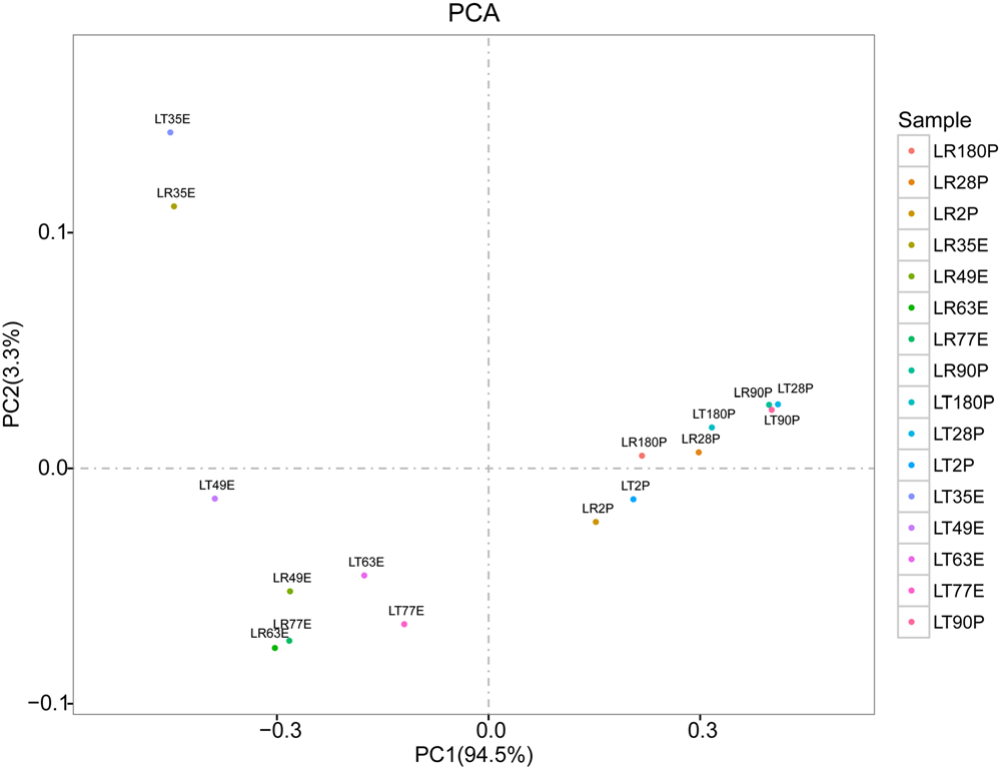
Category DE-miRNA expression pattern. PCA analysis of DE-miRNAs. The analyses were based on the normalized expression data for DE miRNAs in 8 libraries. The clustering of miRNAs into the pre- and post-natal stages can be clearly observed in the graph. E represents embryonic stages and P represents postnatal stages.

PCA analysis was performed using the expression profiles of these DE miRNAs (Fig. 1). Two clusters were found: 1) embryonic stages of LR and LT (in which 35 dpc was a subgroup); and 2) postnatal stages of both breeds. Briefly, the same stages in LR and LT could be classified into the same clusters, but with some differences in most cases, indicating that the main distinctions in the miRNA expression profiles occurred in the embryonic and postnatal stages. Nevertheless, there were still notable differences between pig breeds at the same stage, such as at 35 dpc, 49 dpc, 63 dpc, and 2 dpn. Thus, to simplify the analysis of the differences between the breeds, we grouped the embryonic stages (35, 49, 63, and 77 dpc) into group 1 (G1) and the postnatal stages (2, 28, 90, and 180 dpn) into group 2 (G2).

**Figure 2:**
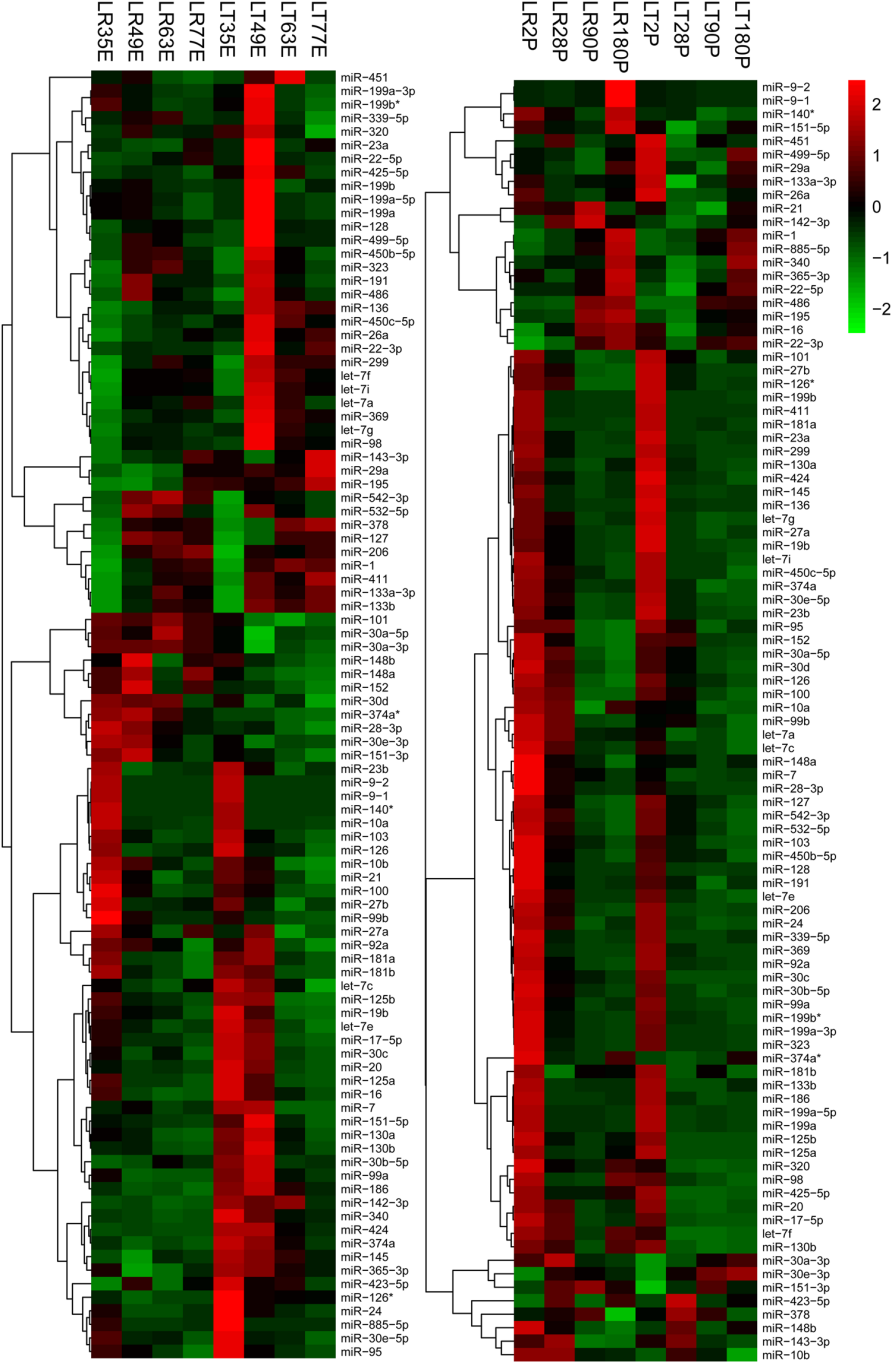
Expression pattern analysis of most abundant miRNAs at G1 and G2 in both breeds. The figure shows heatmaps of DE miRNAs between LR and LT at G1 (95 miRNAs, left) and G2 (96 miRNAs, right). The highest to lowest fold changes are marked from red to green.

In general, miRNAs with higher expression levels play vital roles. To understand the expression characteristics of abundant miRNAs in LR and LT at G1 and G2, heatmaps of the most abundant miRNAs at G1 (95 miRNAs) and G2 (96 miRNAs) were generated, as shown in Fig. 2. In brief, more than two-thirds of the miRNAs with distinct expression patterns between breeds were observed across several stages. In the G1 group, 35 and 49 dpc showed notable, differences in the miRNA expression profiles between breeds, of which the lard-type LT had more highly expressed miRNAs. In the G2 group, however, the lean LR had higher miRNA expression level, though the expression profiles of most miRNAs were similar between breeds in this group. Interestingly, the expression patterns of myogenic miRNAs (miR-1, 206 and 133) also showed distinct between-breed differences in G1 and G2.

### DE-miRNAs between breeds at the same stages

In comparing the DE-miRNAs between LR and LT at 8 stages, we identified miRNAs with a fold change ≥ 2 and a P value < 0.05 as significantly DE miRNAs. In the following results, up-regulated miRNAs means higher expression and down-regulated miRNAs means lower expression in LR than that in LT, respectively. In the embryonic stages, more down-regulated miRNAs than up-regulated miRNAs were found in LR. In the postnatal stages, however, more up-regulated miRNAs than down-regulated miRNAs were observed in LR except at 2 dpn, when nearly an equal amount of up- and down-regulated miRNAs were found in LR compared with LT (Table 1, Tables S3 and S5). These findings further supported the results of PCA analysis showing that G1 and G2 group had distinct miRNA expression patterns.

**Table 1:** Summary of significantly DE-miRNAs between breeds by stage and group.

	No. of up-regulated miRNAs	No. of down-regulated miRNAs	Total no. of DE-miRNAs
LR-LT (35E)	8	45	53
LR-LT (49E)	12	65	77
LR-LT (63E)	4	8	12
LR-LT (77E)	6	9	15
LR-LT (2P)	11	12	23
LR-LT (28P)	40	6	46
LR-LT (90P)	27	7	34
LR-LT (180P)	47	11	58
G1	23	81	104
G2	94	30	124

E represents embryonic stages and P represents postnatal stages.

**Figure 3:**
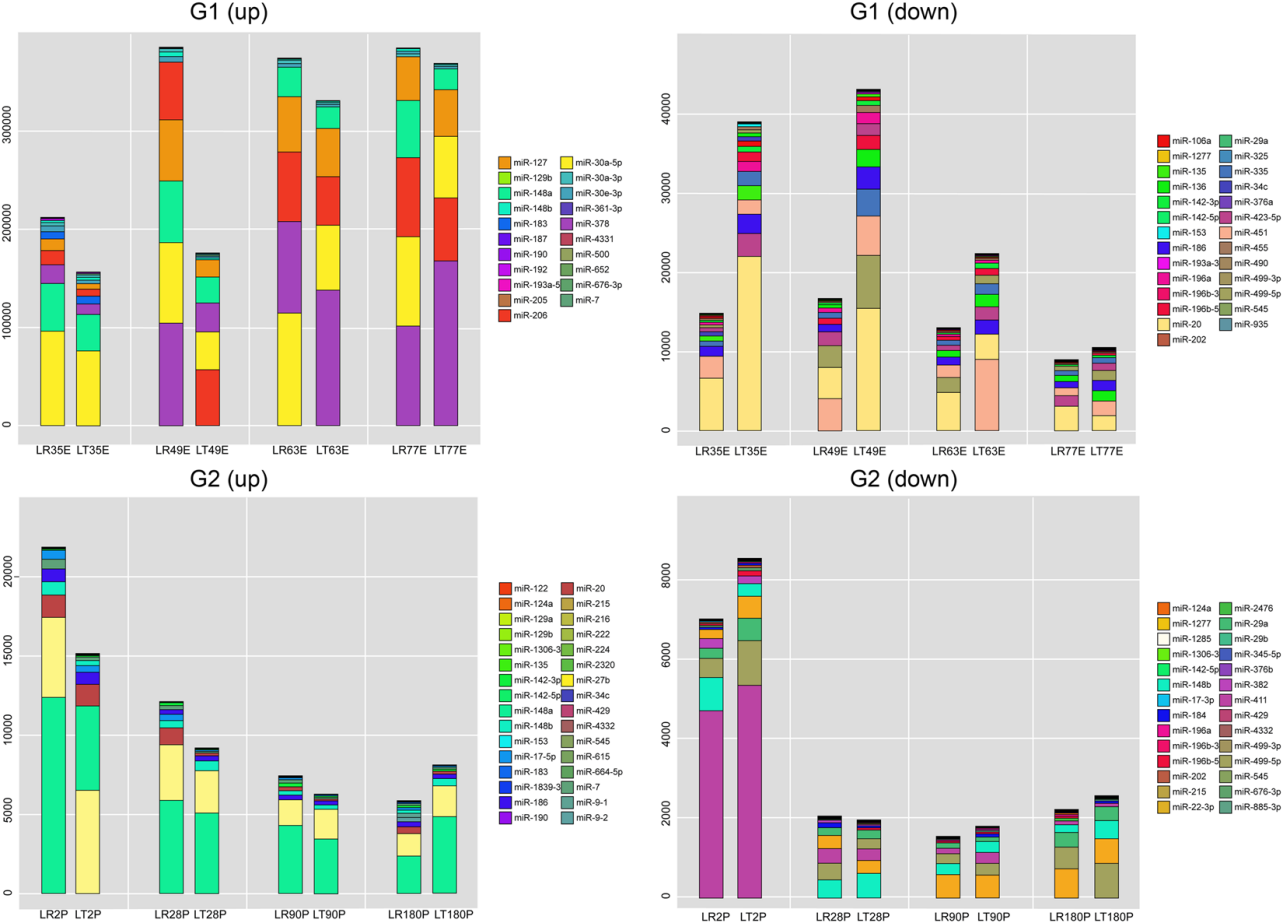
Summary of the most abundant up- and down-regulated miRNAs between breeds. (**A**) most abundant up-regulated miRNAs in G1; (**B**) most abundant down-regulated miRNAs in G1; (**C**) most abundant up-regulated miRNAs in G2; and (**D**) most abundant down-regulated miRNAs in G2. Both breeds are depicted in (**A**–**D**). Different colors representing different miRNAs are listed on the right.

To understand which DE-miRNAs were abundantly expressed in the four subgroups, the top 10 most abundant DE-miRNAs across four stages in each subgroup were compared between breeds; the results are graphed in Fig. 3. From only these most abundant miRNAs, obvious differences were found between breeds in both the prenatal (35, 49 and 63 dpc) and postnatal (2 dpn) stages, which are consistent with the PCA results and the heatmaps. From these abundantly expressed DE-miRNAs, miR-30a showed higher expression in LR than in LT, especially at 49 dpc. In a comparison between the pig breeds, 49 dpc showed the most significant differences in G1 (up-regulated), in which miR-378, miR-30a, miR-148a, and miR-127 showed drastic changes. Moreover, the myogenic miRNA miR-206 also showed a higher expression level in LR and distinctly different distinct expression patterns between breeds from 49 to 77 dpc. In G1 (down-regulated), the stages from 35 to 63 dpc showed the most significant differences between breeds, in which miR-20, miR-499, miR-451 and miR-335 had drastic changes. In G2 (up-regulated), miR-148a was markedly the most different between breeds at 2 dpn. In G2 (down-regulated), no obviously distinct miRNA was found between breeds. Briefly, these findings not only revealed a number of candidate miRNAs with potential roles in regulating muscle development but also identified critical stages at which these miRNAs function.

**Figure 4:**
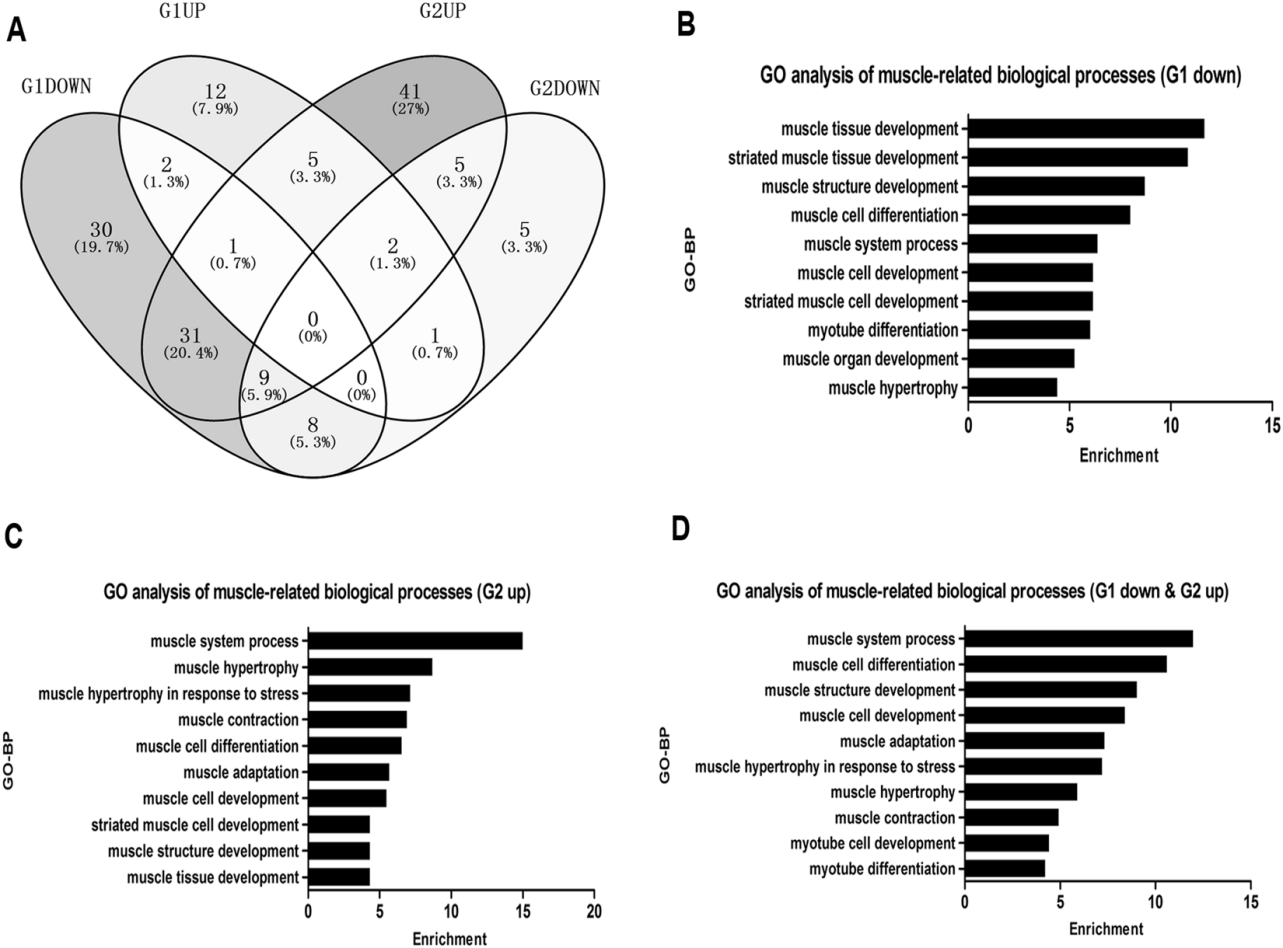
Venn diagram and GO analysis of DE-miRNAs. (**A**) G1 represents embryonic stages, G2 represents postnatal stages, the upper circle represents miRNAs with higher expression in LR, and the lower circle represents miRNAs with lower expression in LR in at least one stage. (**B**–**D**) Results of GO analysis based on G1 (down) (**B**), G2 (up) (**C**) and their shared miRNAs (**D**).

To discover the functions of miRNAs in muscle development, we classified all the DE-miRNAs (Table S4) into the following four subgroups: G1 (down), G1 (up), G2 (down), and G2 (up). A Venn diagram showed that G1 (down) and G2 (up) had the largest number of DE-miRNAs as well as the largest number of shared DE-miRNAs (Fig. 4A). Thus, we conducted a GO analysis to find out which biological progresses (BPs) these DE-miRNAs participated in. Myogenic BPs are listed in Fig. 4. Notably, in G1 (down) group, most enriched BPs were involved in muscle development, followed by muscle differentiation (Fig. 4B), whereas in G2 (up), muscle hypertrophy was the second most enriched BP following muscle system process. Notably, approximately half of the DE-miRNAs were shared between G1 (down) and G2 (up), indicating that many myogenic miRNAs were not only involved in embryonic muscle development but also in muscle hypertrophy after birth.

### Significantly enriched expression trends of miRNA target genes showed distinct between breeds in the transcriptomic data

**Figure 5:**
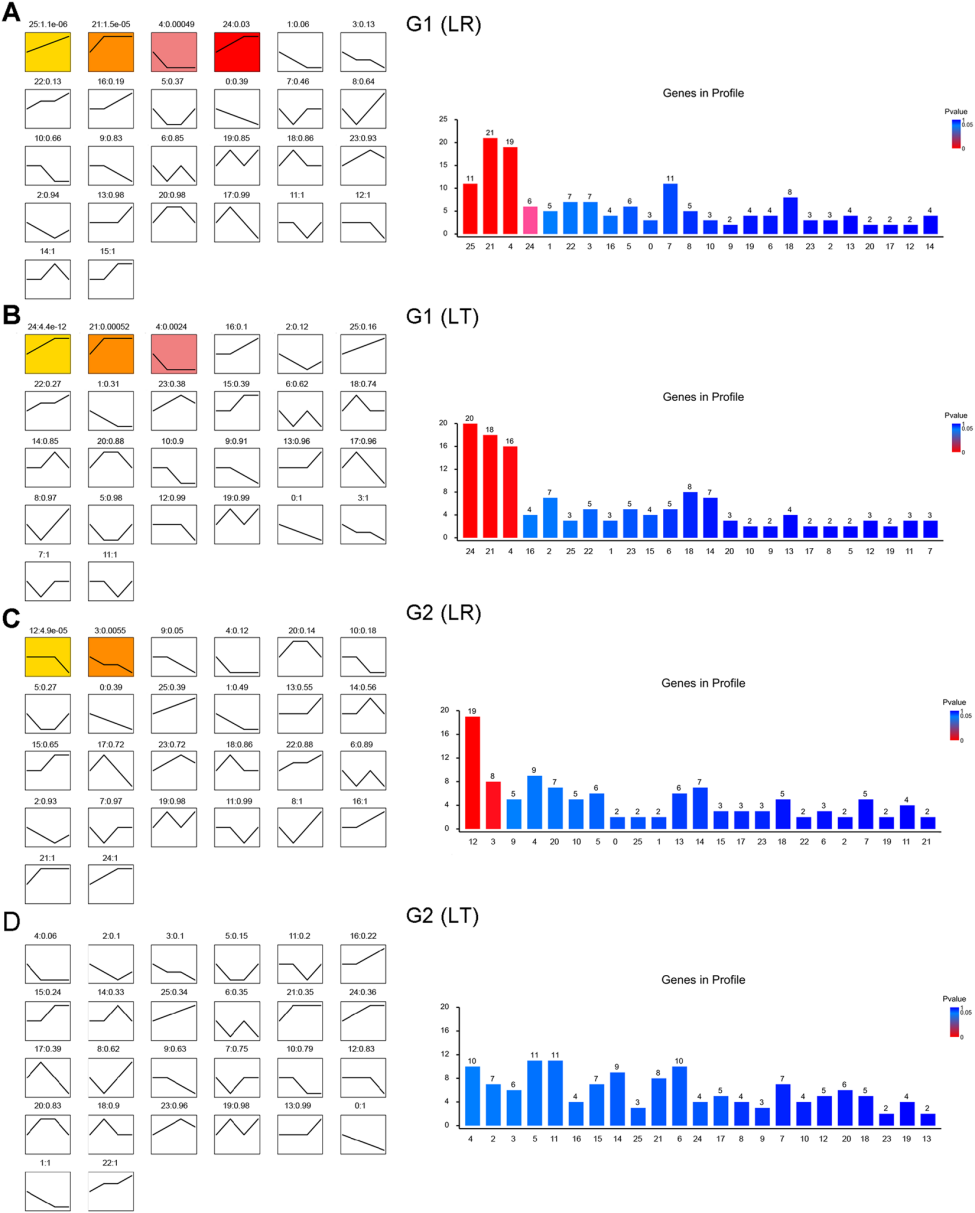
STEM analysis of gene expression profiles. (**A**–**D**) Each box indicates a model expression profile; the colored profiles represent profiles that reached statistical significance. The numbers in the box provide the order of the profile (upper left) and the P-value indicating significance (upper right). Histograms on the right show the number of genes involved in each expression profiles; and the lowest to highest P-values are marked from red to blue in the graph.

The myogenic miRNAs that were differentially expressed between breeds at the same stage might be important candidate miRNAs for future studies concerning muscle differentiation and hypertrophy. Therefore, we predicted the target genes of these myogenic miRNAs (Fig. 4B and C) by Targetscan, as previously described^16^. The default parameters of the TargetScan software were as follows: starting from 5′, 2–8 nt small RNA sequences were chosen as the seed sequences to predict the 3′-UTR of transcripts. In total, 203 predicted genes were selected (Table S6). The Short Time-series Expression Miner (v.1.1, STEM) was then used to cluster the temporal gene expression profiles of LR and LT in the G1 and G2 groups. In the G1 (LR) group, four expression profiles showed that 57 of 203 genes were significantly enriched and tended to be up-regulated or down-regulated during at least one stage (Fig. 5A). In the G1 (LT) group, however, the 25^th^ expression trend was not significantly enriched, although the other three expression trends (24, 21 and 4) were maintained. In the G2 (LR) group, 27 of 203 genes were found to be enriched in two expression profiles (Fig. 5C), whereas in the G2 (LT) group, no gene were found significantly enriched (Fig. 5D). These results suggested that the expression profiles of these target genes were not identical between breeds and may have influenced muscle development.

### Interaction network analysis of miRNAs and targets predicted by GO to be involved in myogenic-related BPs

**Figure 6:**
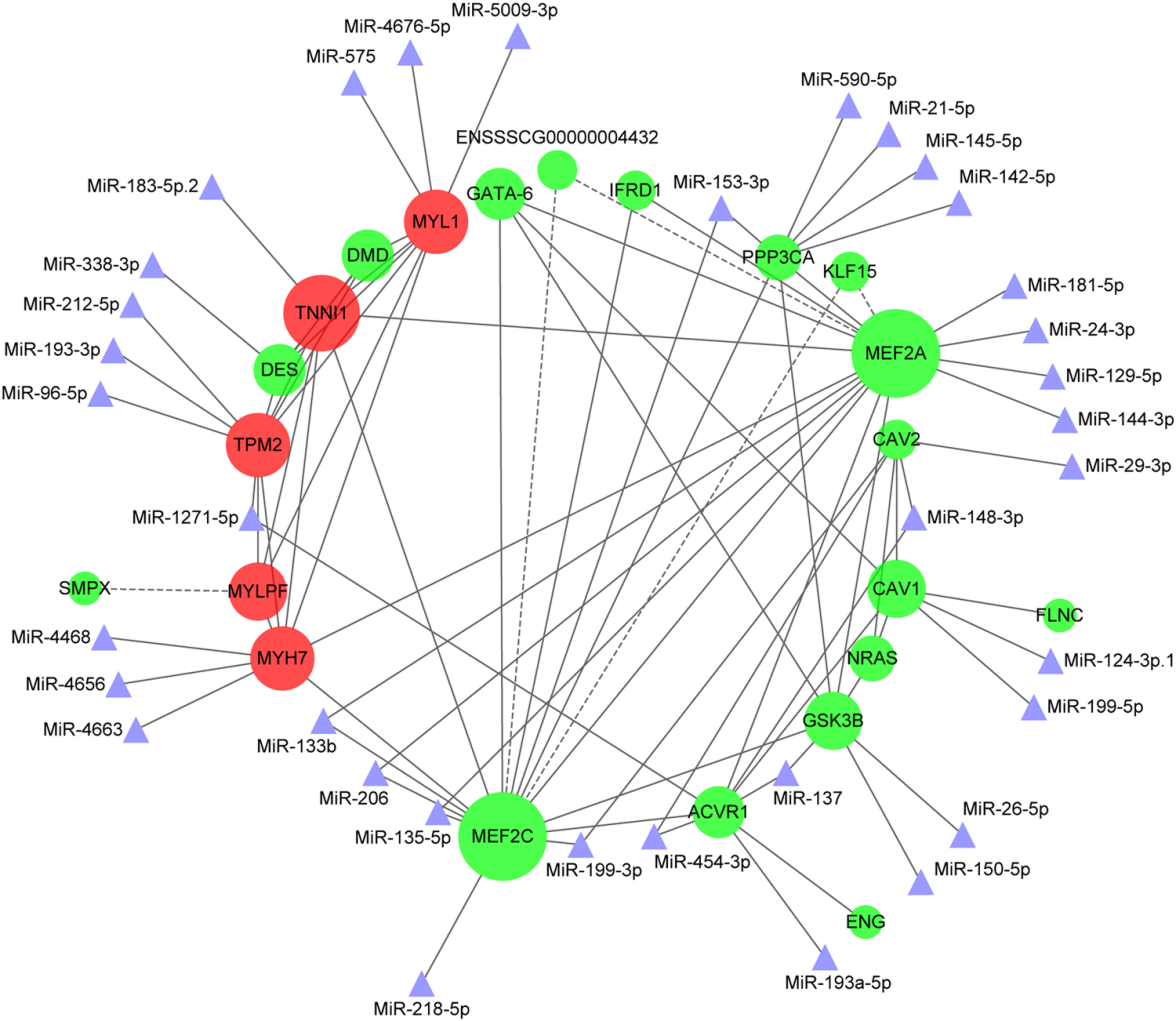
Interaction network of miRNAs and genes involved in significantly enriched STEM expression profiles. Interaction scores were predicted by STRING analysis and illustrated using Cyotoscape software. A larger cycle represents a stronger interaction. The strongest to weakest interaction are marked in red to blue. Genes are drawn in a cycle, while miRNAs are drawn in a triangle. Validated interactions are depicted by solid lines, and predicted interactions are marked by dotted lines.

To further validate the interaction mechanisms of genes predicted by GO to have myogenic-related BPs, we performed a network analysis of miRNAs and their 138 target genes involved in all the significantly enriched profiles resulting from STEM analysis in G1 and G2 of both breeds (Table S8). The STRING website was used to predict the interaction score of candidate genes, and a total of 48 interaction scores were listed (Table S9). Then, Cytoscape software was performed to visualize the data. The interaction of 22 targets and 35 miRNAs were observed in the network (Fig. 6). Some of miRNAs showed multiple strong interactions with other genes; some of these interactions included well-known, crucial myogenic miRNAs and genes (miR-206, miR-133b, miR135-5p, MEF2A, MEF2C, etc.) and myofibrillar genes (MYH7, TPM2, DES, etc.). Thus, it can be reasonably speculated that myogenic miRNAs/genes may interact with myofibrillar genes in muscle development during embryonic stages and muscle hypertrophy during postnatal stages in both LR and LT.

## Discussion

In this study, we conducted an integrative analysis of microRNA-mRNA expression profiles for LR and LT during 8 skeletal muscle developmental stages, including embryonic and postnatal stages, using the Solexa sequencing method. We discovered distinct miRNA expression profiles between breeds with different muscle production, provided new data on the muscle development regulatory network. We also found several miRNAs/genes that may cooperate with each other to regulate secondary myofiber differentiation during the embryonic stages and muscle hypertrophy during the postnatal stages.

### Embryonic and postnatal stages had distinct miRNA expression patterns in both breeds

PCA analysis of these DE-miRNAs showed that embryonic stages and postnatal stages were well separated into two clusters, in which the same stages in LR and LT could be classified into same clusters in most cases. This result indicated that the distinctions in the miRNA expression profiles are larger between embryonic and postnatal stages within breeds than between breeds.

### Comparison of significantly DE-miRNAs between breeds at the same stages

Compared with LT, LR showed more down-regulated miRNAs than up-regulated miRNAs in the embryonic stages; by contrast, LR showed more up-regulated miRNAs in the postnatal stages. The stage of secondary myofiber formation occurs in pigs at 49 to 90 dpc. In our study, a large number of miRNAs were down-regulated in LR, leading to the expression of their target genes to promote muscle differentiation and form more secondary myofibers. LT, however, showed fewer down-regulated miRNAs and thus less intense secondary myofiber formation.

Notably, though both breeds showed more similarities at the same stages in most cases, there were still differences at 35, 49, 63, and 77 dpc and 28 dpn. Indeed, previous studies have shown that pig primary myofibers form between 35 and 55 dpc, followed by the formation of secondary myofibers around each primary myofiber between 50 and 90 dpc. From late gestation until the first four postnatal weeks, myofibers undergo a maturation process^10^. Our previous research showed that in LT, myogenesis started earlier but progressed more slowly than in LR. Moreover, the stages ranging from 49 dpc to 77 dpc are critical for the formation of different muscle phenotypes. Interestingly, the 95 most abundant miRNAs showed significantly distinct expression levels at 35 and 49 dpc, further indicating that the embryonic stages, especially the early embryonic stages, determine different muscle development patterns between breeds. These findings were all in line with the previous study, showing that the regulatory mechanisms involved in critical stages before birth may differ between pig breeds^1, 5^.

Abundantly expressed miRNAs play more functions. Thus, several abundantly up- and down-regulated miRNAs at all stages were compared between breeds. miR-186, which inhibits muscle cell differentiation through the down-regulation of myogenin^17^, was found to be down-regulated in LR during the embryonic stages. In line with the phenomenon that LR showed intense secondary myofiber formation, the reduced expression of miR-186 may have benefited muscle differentiation in LR. After birth, however, the expression of miR-186 showed no difference between breeds, suggesting that miR-186 may not participate in muscle hypertrophy. miR-206 promotes the differentiation of skeletal muscle cells through the inhibition of histone deacetylase 4(HDAC4) which controls muscle differentiation^18^. Thus, miR-206 plays an opposite role to miR-186 and showed higher expression in LR than in LT across the studied embryonic stages (G1 up). Though miR-206 was not selected as a DE miRNAs, as it failed to meet the selection criteria, this miRNA indeed showed distinct expression patterns between breeds. Figure 3 shows that in G1 (up), we clearly found that 49 dpc showed the most significant differences between breeds; in this group, miR-378, miR-148a and miR-127 showed drastic changes. By contrast, in G1 (down), 35–63 dpc showed the most significant differences between breeds; in this group, miR-20, miR-451, miR-499 and miR-335 showed drastic changes. Myofiber formation progresses from 35 to 77 dpc. miR-378 prevents cardiomyocyte hypertrophy through the repression of four components of the MAP kinase pathway^19^. A trans-homologous interaction between miR-127 and RTL1 was previously found in mouse muscle development^20^, and RTL1 was investigated as a candidate gene in postnatal skeletal muscle growth in callipyge lambs^21^. miR-499 is a muscle-specific miRNA and an intronic miRNA of the myosin heavy chain gene MYH14 that plays a key role in muscle fiber-type specification in mammals^22^. These results indicated the roles of these miRNAs in secondary myofiber formation. In G2 (down), the most significant differences between breeds were observed at 2 dpn, when miR-29 and miR-499 showed drastic changes. A lecture showed that the inhibition of miR-29 by TGF-beta-Smad3 signaling through dual mechanisms promotes the trans-differentiation of mouse myoblasts into myofibroblasts^23^. In agreement with a previous study, miR-29a was also increased in abundance after birth^13^. These results indicated these miRNAs play important roles in muscle hypertrophy.

Moreover, miRNAs showed distinct profiles in G1 and more similarity in G2 between breeds, suggesting that the differences in muscle production was mainly determined during G1. These myogenic-related miRNAs most likely play important roles in regulating the distinct differences in muscle production between breeds. As the function of miRNAs during muscle development is currently poorly understood, our findings provided a number of miRNAs that may be involved in the regulatory networks involved in muscle development, especially in terms of between-breed differences. Future studies of these miRNAs are needed.

A Venn diagram showed that in G1 (down), the most enriched BPs were involved in muscle development and differentiation, while in G2 (up), muscle hypertrophy was the second most enriched BP of myogenic-related GO terms. These findings are in agreement with the phenomenon that in G1, pig muscle development mainly occurred in two waves of muscle development and differentiation, whereas in G2, muscle hypertrophy occurred; the miRNAs enriched in these biological processes are very likely to regulate distinct muscle differentiation and hypertrophy processes between breeds.

### Regulatory network of predicted genes involved in a GO analysis of myogenic-related BPs

mRNAs with similar expression patterns might be function-related. Thus, we performed a STEM analysis to cluster the temporal profiles of gene expression of G1 and G2 in LR and LT based on the transcriptomic data obtained from the same samples. In G1 of both breeds, significantly enriched expression profiles were up- and down-regulated in at least one stage, whereas in G2 of LR, the significantly enriched expression profiles were only down-regulated in at least one stage. By contrast, in G2 of LT, no significantly enriched profiles were found. These results suggested that the interactions between miRNAs and their target genes played different roles in G1 and G2 between LR and LT. Since the candidate genes found in significantly enriched profiles may influence muscle development, further study is needed.

To reveal the exact miRNAs and genes that regulate muscle development, we performed a network analysis of 35 miRNAs and their 138 target genes. Among the 35 miRNAs found in the network, miR-133b and miR-206 are known muscle-specific miRNAs that are induced during muscle differentiation and increase the expression of myogenic determination and differentiation factors (such as MEF2A and MEF2C in our network)^24, 25^. Notable, 7 miRNAs were shared by two genes in the network. For example, miR-137 showed an interaction with activin A receptor type 1 (ACVR1) and glycogen synthase kinase 3 beta (GSK3B) in the network, while cross-talk between GSK3B and p38 map kinase (p38MAPK) regulated myocyte enhancer factor 2 (MEF2) activity in skeletal and cardiac muscle^26^.

Among the 22 genes found in the network, the MRFs and MEF2 families are key factors in regulating myogenic processes and differentiation^27–29^. In our study, MEF2A and MEF2C were found to play central roles in regulating other genes in the network (with the largest number of interactions). Several genes showed strong interactions in the network. For example, filamin C (FLNC) has crucial roles in muscle development and the maintenance of muscle structural integrity, and plays a role in the TRIO-FLNc-dependent pathway, which maintains proper myotube structure^30^. Dexamethasone downregulates caveolin-1 (CAV1), causing muscle atrophy via the inhibition of insulin signaling^31^. CAV1 (−/−) and CAV2 (−/−)-deficient mice both display numerous skeletal muscle abnormalities characterized by tubular aggregate formation^32^. GATA binding protein 6 (GATA6) induces differentiation of mesoangioblasts into smooth muscle^33^. Small muscle protein, X-linked (SMPX) plays a role in skeletal muscle hypertrophy^34^, and Protein phosphatase 3 catalytic subunit alpha (PPP3CA) plays a critical role in controlling skeletal muscle fiber type^35^, while abnormal splicing of the penultimate exon of dystrophin (DMD) compromises muscle fiber maintenance in myotonic dystrophy^36^. These well-known genes all play crucial roles in muscle development, differentiation, and hypertrophy.

Many myofibrillar proteins (myosin, troponin, among others) are known to exist as multiple isoforms^37^. Myosin comprises approximately one-third of the total muscle proteins and is known to produce multiple isoforms^5^. In our study, 6 myofibrillar genes were found: troponin I1 (TNNI1), myosin, light polypeptide 1 (MYL1), DES, myosin light chain, phosphorylatable (MYLPF), TPM2 and MYH7. Thus, it can be reasonably speculated that in LR and LT, these known myogenic genes may be distinctly expressed to regulate myofibrillar genes in muscle development during the embryonic stages and muscle hypertrophy during the postnatal stages. A recent study comparing pre- and post-natal muscle microRNA expression profiles in the longissimus dorsi muscle of LR and Pietrain pigs also revealed that several pathways related to muscle development were enriched with both the predicted targets of the differentially expressed miRNAs and the miRNA with breed and/or stage differences identified in the present study^13^. Those findings further indicate that miRNAs could play key roles in the phenotypic variations of porcine muscles. In summary, our study contributes to an understanding of the regulatory mechanisms involved in muscle development and hypertrophy between pig breeds with distinct differences in muscle production.

## Electronic supplementary material

Table S1

Table S2

Table S3

Table S4

Table S5

Table S6

Table S7

Table S8

## References

[1] Zhao X (2011). Comparative Analyses by Sequencing of Transcriptomes during Skeletal Muscle Development between Pig Breeds Differing in Muscle Growth Rate and Fatness. PLoS ONE.

[2] Qin L (2013). Integrative analysis of porcine microRNAome during skeletal muscle development. PloS one.

[3] Li JQ (2003). Genetic effects of IGF-1 gene on the performance in Landrace x Lantang pig resource population. Yi Chuan Xue Bao.

[4] Newcom DW (2004). Breed differences and genetic parameters of myoglobin concentration in porcine longissimus muscle. J Anim Sci.

[5] Zhang X (2016). iTRAQ-based quantitative proteomic analysis reveals the distinct early embryo myofiber type characteristics involved in landrace and miniature pig. BMC Genomics.

[6] Suzuki A (1991). Carcass composition and meat quality of Chinese purebred and European x Chinese crossbred pigs. Meat Sci.

[7] Picard B (2010). Skeletal muscle proteomics in livestock production. Briefings in functional genomics.

[8] Picard B, Lefaucheur L, Berri C, Duclos MJ (2002). Muscle fibre ontogenesis in farm animal species. Reprod Nutr Dev.

[9] Tang, Z. *et al*. LongSAGE analysis of skeletal muscle at three prenatal stages in Tongcheng and Landrace pigs. *Genome Biol* 8 (2007). 10.1186/gb-2007-8-6-r115 PMC2394763 17573972

[10] Glass DJ (2005). Skeletal muscle hypertrophy and atrophy signaling pathways. Int J Biochem Cell Biol.

[11] Nelson P, Kiriakidou M, Sharma A, Maniataki E, Mourelatos Z (2003). The microRNA world: small is mighty. Trends Biochem Sci.

[12] Kloosterman WP, Plasterk RH (2006). The diverse functions of microRNAs in animal development and disease. Dev Cell.

[13] Siengdee P (2015). Pre- and post-natal muscle microRNA expression profiles of two pig breeds differing in muscularity. Gene.

[14] Genovesi LA, Carter KW, Gottardo NG, Giles KM, Dallas PB (2011). Integrated Analysis of miRNA and mRNA Expression in Childhood Medulloblastoma Compared with Neural Stem Cells. PLoS ONE.

[15] Yu, F. *et al*. Recent Advances in Information Technology. *The Scientific World Journal* 2014 (2014). 10.1155/2014/746479 PMC4119702 25110742

[16] Pan Y, Guo Y, Luo Y, Li H, Xu Y (2016). MicroRNA expression profiling of Chinese follicular lymphoma by microarray: A preliminary study. International immunopharmacology.

[17] Antoniou A, Mastroyiannopoulos NP, Uney JB, Phylactou L (2014). A. miR-186 Inhibits Muscle Cell Differentiation through Myogenin Regulation. Journal of Biological Chemistry.

[18] Huang QK (2016). MiR-206 Attenuates Denervation-Induced Skeletal Muscle Atrophy in Rats Through Regulation of Satellite Cell Differentiation via TGF-β1, Smad3, and HDAC4 Signaling. Medical Science Monitor.

[19] Ganesan J (2013). miR-378 prevents cardiomyocyte hypertrophy through repression of four factors in the MAP kinase pathway. Naunyn-Schmiedebergs Arch. Pharmacol.

[20] Allen S, Ito M, Murray A, Ferguson-Smith A (2012). A trans-homologue interaction between miR-127 and Rtl1: roles in mouse muscle development. Genet. Res..

[21] Fleming-Waddell JN (2009). Effect of DLK1 and RTL1 but Not MEG3 or MEG8 on Muscle Gene Expression in Callipyge Lambs. PloS one.

[22] Bhuiyan SS (2013). Evolution of the myosin heavy chain gene MYH14 and its intronic microRNA miR-499: muscle-specific miR-499 expression persists in the absence of the ancestral host gene. BMC Evol. Biol..

[23] Zhou L (2012). Inhibition of miR-29 by TGF-beta-Smad3 Signaling through Dual Mechanisms Promotes Transdifferentiation of Mouse Myoblasts into Myofibroblasts. PloS one.

[24] Kim HK, Lee YS, Sivaprasad U, Malhotra A, Dutta A (2006). Muscle-specific microRNA miR-206 promotes muscle differentiation. J. Cell Biol..

[25] Koutsoulidou A, Mastroyiannopoulos NP, Furling D, Uney JB, Phylactou LA (2011). Expression of miR-1, miR-133a, miR-133b and miR-206 increases during development of human skeletal muscle. BMC Dev. Biol..

[26] Dionyssiou MG (2013). Cross-talk between glycogen synthase kinase 3 beta (GSK3 beta) and p38MAPK regulates myocyte enhancer factor 2 (MEF2) activity in skeletal and cardiac muscle. Journal of Molecular and Cellular Cardiology.

[27] Te KG, Reggiani C (2002). Skeletal muscle fibre type specification during embryonic development. J Muscle Res Cell Motil.

[28] Liu N, Bassel-Duby R (2015). Regulation of skeletal muscle development and disease by microRNAs. Results Probl Cell Differ.

[29] Charge SB, Rudnicki MA (2004). Cellular and molecular regulation of muscle regeneration. Physiol Rev.

[30] Dalkilic I, Schienda J, Thompson TG, Kunkel LM (2006). Loss of FilaminC (FLNc) results in severe defects in myogenesis and myotube structure. Mol Cell Biol.

[31] Son YH (2015). Dexamethasone downregulates caveolin-1 causing muscle atrophy via inhibited insulin signaling. J Endocrinol.

[32] Schubert W (2007). Caveolin-1(−/−)- and caveolin-2(−/−)-deficient mice both display numerous skeletal muscle abnormalities, with tubular aggregate formation. Am J Pathol.

[33] Donati C (2011). Sphingosine 1-Phosphate Induces Differentiation of Mesoangioblasts towards Smooth Muscle. A Role for GATA6. PloS one.

[34] Kemp TJ (2001). Identification of a novel stretch-responsive skeletal muscle gene (Smpx). Genomics.

[35] Wan L, Ma JS, Xu GY, Wang DH, Wang NL (2014). Molecular Cloning, Structural Analysis and Tissue Expression of Protein Phosphatase 3 Catalytic Subunit Alpha Isoform (PPP3CA) Gene in Tianfu Goat Muscle. International journal of molecular sciences.

[36] Rau F (2015). Abnormal splicing switch of DMD’s penultimate exon compromises muscle fibre maintenance in myotonic dystrophy. Nature communications.

[37] Schiaffino S, Reggiani C (1996). Molecular diversity of myofibrillar proteins: gene regulation and functional significance. Physiol Rev.

